# How to Tackle Tremor – Systematic Review of the Literature and Diagnostic Work-Up

**DOI:** 10.3389/fneur.2012.00146

**Published:** 2012-10-23

**Authors:** A. W. G. Buijink, M. F. Contarino, J. H. T. M. Koelman, J. D. Speelman, A. F. van Rootselaar

**Affiliations:** ^1^Department of Neurology and Clinical Neurophysiology, Academic Medical Center, University of AmsterdamAmsterdam, Netherlands

**Keywords:** tremor, essential tremor, diagnosis, electromyography, differential diagnosis, action tremor

## Abstract

**Background:** Tremor is the most prevalent movement disorder in clinical practice. It is defined as involuntary, rhythmic, oscillatory movements. The diagnostic process of patients with tremor can be laborious and challenging, and a clear, systematic overview of available diagnostic techniques is lacking. Tremor can be a symptom of many diseases, but can also represent a distinct disease entity. **Objective:** The objective of this review is to give a clear, systematic and step-wise overview of the diagnostic work-up of a patient with tremor. The clinical relevance and value of available laboratory tests in patients with tremor will be explored. **Methods:** We systematically searched through EMBASE. The retrieved articles were supplemented by articles containing relevant data or provided important background information. Studies that were included investigated the value and/or usability of diagnostic tests for tremor. **Results:** In most patients, history and clinical examination by an experienced movement disorders neurologist are sufficient to establish a correct diagnosis, and further ancillary examinations will not be needed. Ancillary investigation should always be guided by tremor type(s) present and other associated signs and symptoms. The main ancillary examination techniques currently are electromyography and SPECT imaging. Unfortunately, many techniques have not been studied in large prospective, diagnostic studies to be able to determine important variables like sensitivity and specificity. **Conclusion:** When encountering a patient with tremor, history, and careful clinical examination should guide the diagnostic process. Adherence to the diagnostic work-up provided in this review will help the diagnostic process of these patients.

## Introduction

Tremor is defined as rhythmic, oscillatory involuntary movements (Deuschl et al., 1998). It is a common symptom of a wide range of neurological and other disorders, as well as a disease entity in itself. The diagnostic process of patients with tremor can be laborious and difficult. For example, 30 to even 50% of patients with essential tremor are misdiagnosed (Jain et al., 2006). Sometimes, effective specific treatment fails or is delayed due to limited diagnostic tools. Even if treatment is successful, the therapeutic process could take months, involving different drugs, with the consequence of unnecessary side-effects of medication. This process can also be costly, since wrong diagnostic tests and wrong types of medication may be used. The objective of this review is to provide a clinical practice guideline with respect to diagnosing tremor disorders. Different types of tremor, their typical clinical features and underlying pathophysiology are summarized. A systematic literature search has been performed on the diagnostic use of ancillary examinations in the light of differentiating tremor syndromes. Finally, we propose a flow chart on how to approach a patient presenting with tremor.

## Methods

We searched EMBASE for identifying all articles on diagnostic techniques in tremor using the following search: (exp tremor/OR tremor$.tw.) AND [exp electromyography (EMG)/OR electromyograph*.tw. OR EMG.ti,ab. OR (tremor adj2 registration).tw. OR accelerometer/or accelerometry/OR exp electroencephalography/OR EEG.ti,ab. OR electromyograph$.ti,ab. OR ioflupane i 123/OR datscan.mp. OR exp nuclear magnetic resonance imaging (MRI)/OR (magnetic adj resonance).ti,ab. OR MRI.ti,ab.] AND (exp diagnosis/OR di.fs. OR diagnos*.ti,ab) NOT (“review”/OR case report/) NOT (animal/not human/). This search specifically looked for studies on EMG, accelerometry, electroencephalography, [^123^I]-FP-CIT single photon emission computerized tomography, and MRI. Furthermore, the search included studies on tremor in combination with “diagnosis.”

The search retrieved 2114 citations. Reviews, case-reports, animal studies, studies on therapy or studies on pathophysiology were excluded, leaving a remaining 425 articles, 40 of these articles were included in this review, because these studies investigated the value and/or usability of a certain diagnostic test for clinical practice in patients with tremor and were available in English. The 40 articles included in this review were supplemented by an additional 49 articles. These articles were identified as containing relevant data while reviewing references cited in the retrieved articles, or provided important background information.

## Results

### Classification of tremor

Tremor can be present during rest (rest/resting tremor), or during voluntary contraction of muscles (action tremor; Table 1). Action tremor can be further divided into several subtypes, summarized in Table 1 (Deuschl et al., 1998). Tremor can also be categorized by frequency. Three frequency domains have been appointed: (1) low frequency tremors with a frequency below 4 Hz, (2) middle frequency tremor between 4 and 7 Hz, and (3), tremors with a high-frequency above 7 Hz (Deuschl et al., 1998).

**Table 1:** **Types of tremor**.

	Subtype	Occurrence	Physical examination
**TYPES OF TREMOR**			
Rest tremor	Rest/resting tremor	In a body part that is not voluntarily activated and completely supported against gravity	Letting forearms rest on legs or armrest, flexed elbows, with palms in a supinated position
Action tremor	Postural tremor	During voluntarily maintaining a position against gravity	Keep arms and fingers in stretched and flexed positions
	Simple kinetic tremor	During non-target-directed movements	E.g., finger tapping
	Intention tremor	During visually guided movements toward a target at the endpoint of a movement	E.g., finger-to-nose test
	Task-specific kinetic tremor	During a specific skilled task	Specific and aspecific tasks
	Isometric tremor	During isometric muscle contraction	E.g., contraction against a static object, making a fist
	Isometric orthostatic tremor	During stance or stance phase of walking	Standing, walking

### The patient with tremor

The prevalence of a specific tremor disorder defines the prior probability of encountering a patient with that tremor disorder. Every tremor disorder has certain characteristics that can help to differentiate from other tremor disorders, such as age at onset, sequence of spread, sudden, or gradual onset and the body part(s) first affected (Bain, 2007). For an overview of most common tremor disorders, see Table 2.

**Table 2:** **Most common tremor disorders**.

Diagnosis	Tremor type(s)	Frequency range	Accompanying features	Pathophysiology
**MOST COMMON TREMOR DISORDERS**				
Enhanced physiologic tremor	Posture	5–12 Hz	Increases after caffeine intake, and upon stress and anxiety	Consists of two distinct oscillations, a mechanical-reflex oscillations and a central-neurogenic oscillation (Elble, 1996)
Essential tremor	Posture intention rest	4–12 Hz	Additional or isolated head tremor (Critchley, 1949), tandem gait abnormalities (Stolze et al., 2001)	Involvement of parts of the cerebello-thalamo-cortical network (Louis, 2011)
Parkinsonian tremor	Rest posture intention	4–9 Hz	Bradykinesia, rigidity, postural problems	Degeneration of dopaminergic pathways (Kraus et al., 2006)
Dystonic tremor	Posture intention rest	4–10 Hz	“Gestes antagonistes,” dystonic posturing of other body parts (Deuschl et al., 1998)	Unknown, but can be related to basal ganglia dysfunction observed in dystonia (Pont-Sunyer et al., 2012)
Psychogenic tremor	Rest posture intention	4–12 Hz	Entrainment, increase in tremor amplitude with loading, inconsistent over time (Edwards and Schrag, 2011)	Unknown (Edwards and Schrag, 2011)
Toxic and drug-induced tremor	Posture intention rest	3–12 Hz	Medication/drug use, exposure to heavy metals, symptoms of metabolic disorders (Puschmann and Wszolek, 2011)	Various mechanisms (Morgan and Sethi, 2005)
Cerebellar tremor	Intention	2–5 Hz	Eye-movement abnormalities, dysmetria, dyssynergia, trunk titubation (Degardin et al., 2012)	Lesions of the lateral cerebellar nuclei, the superior cerebellar peduncle, or the pathways where they are involved (Pont-Sunyer et al., 2012)
Task-specific tremor	Posture intention	4–8 Hz	Occurs during specific task (i.e., writing; Bain, 2011)	May be related to essential tremor or dystonia (writer’s cramp; Bain, 2011)
Holmes’ tremor	Rest intention posture	2–5 Hz	Evidence of lesions of the central nervous system (Deuschl et al., 1998), neurological signs associated with lesions	Lesions in the dopaminergic nigrostriatal and cerebello-thalamic pathways (Seidel et al., 2009)
Cortical myoclonic tremor	Posture intention	6–20 Hz	(Family) history of epileptic seizures (van Rootselaar et al., 2006)	GABA_A_-ergic dysfunction within the cerebral cortex (van Rootselaar et al., 2007)
Neuropathic tremor	Posture	4–12 Hz	Muscle weakness, absent reflexes, glove/stocking sensory deficits (Pont-Sunyer et al., 2012)	Slow nerve conduction increases the delay of a stretch reflex response, leading to enhancement of the tremor, but central components can also be involved (Pont-Sunyer et al., 2012)

*An overview of most common tremor disorders. The tremor type(s) and frequency range columns are adapted from the MDS consensus statement (Deuschl et al., 1998)*.

Essential tremor is the most common form of tremor, with an estimated prevalence between 0.4 and 0.9% in the general population, and an increase with age, with a prevalence of up to 4.6% in people over 65 years old, and even 22% in people over 95 years old (Louis and Ferreira, 2010). The mean age at onset of ET is around 45 years, but tremor can also present itself in early adulthood and even during childhood. The incidence increases with advancing age. Usually, patients do not seek medical attention until more advanced age because of its slowly progressive nature. Symmetrical postural and/or intention tremor between 4 and 12 Hz in the arms without any other neurological signs is most suggestive for ET (Deuschl et al., 1998). Tremor in many ET patients attenuates upon alcohol intake (Koller and Biary, 1984). See Table 3 for the diagnostic criteria of ET and the differential diagnosis of middle frequency postural tremor. It has been suggested that intention tremor is more severe than postural tremor, which may even be absent (Brennan et al., 2002). Upper limbs are affected in about 95% of patients, followed by head (34%), lower limbs (20%), voice (12%), face and trunk (5%; Elble, 2000). Rest tremor is present in about 18% of ET patients (Cohen et al., 2003). Task-related disability, such as difficulties with eating and drinking, is indicative for ET. In some cases, ET patients show an autosomal dominant inheritance pattern, with a positive family history ranging from 17 to 100% of the cases, depending on the study (Deng et al., 2007).

**Table 3:** **Clinical criteria for ET and differential diagnosis for a patient with middle frequency postural tremor**.

Clinical criteria for ET (MDS consensus statement Deuschl et al., 1998):	Differential diagnosis middle frequency postural tremor:
*Inclusion criteria*	Essential tremor
Bilateral, largely symmetric postural, or kinetic tremor involving hands and forearms that is visible and persistent	Parkinson’s disease
Additional or isolated tremor in head but absence of abnormal posturing	Enhanced physiologic tremor
*Exclusion criteria*	Dystonic tremor
Other abnormal neurological signs (especially dystonia)	Wilson disease
Presence of known causes of enhanced physiologic tremor	Primary writing tremor
Historical or clinical evidence of psychogenic tremor	Epilepsia partialis continua
Convincing evidence of sudden onset or step-wise deterioration	Familial cortical tremor
Primary orthostatic tremor	Spinal segmental myoclonus
Isolated voice, tongue, chin, leg tremor	Progressive myoclonic ataxia
Isolated position- or task-specific tremor	Spinocerebellar ataxias
	Neuropathic tremor
	Drug-induced tremor
	Metabolic alterations
	Fragile-X-associated tremor/ataxia syndrome (FXTAS)

When a 4- to 9-Hz resting tremor, or “pill-rolling” tremor, typical for Parkinson’s disease (PD) is present, attention should be directed to the presence of rigidity and bradykinesia. The prevalence is roughly estimated to be about 0.3% of the general population, and increases up to 1% in people at the age of 60 (de Lau and Breteler, 2006). Rest tremor in PD usually starts after the age of 60 and progresses gradually. Typical for rest tremor in PD is re-emerging tremor: tremor that is present during rest, disappears upon stretching of the arms, and “re-emerges” when the arms are maintained in the same position. Postural tremor is present in up to 60% of PD patients, and can have a higher tremor frequency (>1.5 Hz) then the rest tremor (Bain, 2007). Cog wheeling can be a phenomenon of both ET and PD, because it appears to be related to the presence of tremor rather than to rigidity (Louis, 2011). For this reason it should not be considered as a differential sign between ET and PD. While a positive response to alcohol intake is in line with the diagnosis of ET, alcohol has no effect on tremor in PD (Koller and Biary, 1984; Lakie et al., 1994; Bain, 2011).

Enhanced physiologic tremor is a high-frequency (8–12 Hz), low-amplitude, mostly postural, bilateral tremor. Drugs and toxins, such as caffeine, induce this form of tremor. Also, tremor intensifies with anxiety, stress, and after strenuous exercise. Enhanced physiologic tremor does not interfere with daily activities, in contrast to ET. Intention tremor is not typical for enhanced physiologic tremor (Deuschl et al., 1998).

Dystonic posturing in the same body part suggests a dystonic tremor, for example cervical dystonia and head tremor. When the trembling body part is not affected by dystonia, but dystonic posturing occurs in other body parts, this is referred to as “tremor associated with dystonia” (Deuschl et al., 1998). Whether this last group should be classified as ET with dystonia or as a “tremulous dystonia syndrome” remains controversial (Schiebler et al., 2011). Usually dystonic tremor increases in amplitude when moving in opposition to the direction of dystonic contractions and tends to show much greater right–left asymmetry than essential tremor (LeDoux, 2012). Some patients have a trick to alleviate tremor, a so-called “sensory trick”. This can be a sign of dystonia (Masuhr et al., 2000). Dystonic tremor occurs usually in patients younger than 50 years. In patients with arm tremor including a resting tremor and reduced arm swing on the affected side, it can be difficult to differentiate between PD and dystonia at an early stage (Schneider et al., 2007). In these cases, attention should be given to other clinical signs of PD or dystonia. Response to levodopa treatment is highly suggestive of PD (D’Costa et al., 1991).

Systemic signs of hyperthyroidism, such as excessive sweating, palpitations, and weight loss, should be checked, since hyperthyroidism can cause a low-amplitude, middle-to-high-frequency postural tremor (Milanov and Sheinkova, 2000). Other metabolic disorders that can cause tremor include renal failure, hypoglycemia, and liver disease (Pont-Sunyer et al., 2012).

In addition to previously mentioned tremor disorders, there are many other, less prevalent, tremor syndromes, some of which should not be missed because they might be treatable (see Table 4 for alarm symptoms in tremor patients). In patients before the age of 40 presenting with tremor, concerns should be raised for Wilson disease, an autosomal recessive inherited disorder in copper-metabolism (Roberts and Schilsky, 2008). Tremor in Wilson disease is often postural, starts in one limb, and may eventually spread to the whole body. “Wing beating tremor” is one of the characteristic symptoms of Wilson disease and consists of a proximal tremor of high amplitude, best seen when the patients stretches the arms (Roberts and Schilsky, 2008; Puschmann and Wszolek, 2011). Patients suspected of having Wilson disease, should be examined for Kayser–Fleischer rings and hepatosplenomegaly (Roberts and Schilsky, 2008). Ancillary examination up to 55 years of age is necessary to exclude or confirm Wilson disease (see Ancillary Examinations) and neuroimaging is indicated in all patients with Wilson disease presenting with neurological symptoms. Wilson disease is not excluded in individuals over 40 years of age, and further evaluation should be carried out when symptoms of Wilson disease are present (Roberts and Schilsky, 2008).

**Table 4:** **Red flags in patients with tremor**.

Red flags in patients with tremor
Unexplained tremor in patient younger than 55
One-sided tremor (not PD)
Sudden onset
Start/change of medication
Other unexplained symptoms

Many types of medication and life-style drugs are known to cause or exacerbate tremor, and therefore, a detailed history of medication use is crucial. The temporal relation of the tremor to the start of medication and the dose-response relationship between increasing the dosage and a simultaneous increase of the tremor should be clarified. Table 5 provides an overview of drugs often involved with action tremor (Morgan and Sethi, 2005). In most instances, drug-induced tremor reduces or even abates after removal of the agent (Morgan and Sethi, 2005). Occupational exposure to heavy metals, such as lead, manganese, and mercury, can induce action and rest tremor. In some patients, tremor remains after withdrawal of heavy metal contact (Urban et al., 1996; Milanov and Kolev, 2001; Bose-O’Reilly et al., 2010). Patient should finally be screened for alcohol abuse, since alcohol overuse and withdrawal can cause tremor.

**Table 5:** **Drugs related to postural and intention tremor**.

Drug group	Postural tremor	Intention tremor
**DRUGS RELATED TO POSTURAL AND INTENTION TREMOR**		
Antiarrhythmics	Amiodarone, mexiletine, procainamide	–
Antibiotics, antivirals, antimycotics	–	Vidarabine
Antidepressants and mood stabilizers	Amitriptyline, lithium, SSRIs	Lithium
Antiepileptics	Valproic acid	–
Bronchodilators	Salbutamol, salmeterol	Salbutamol, salmeterol
Chemotherapeutics	Tamoxifen, cytarabine, ifosfamide	Cytarabine, ifosfamide
Drugs of misuse	Cocaine, ethanol, MDMA, nicotine	Ethanol
Gastrointestinal drugs	Metoclopramide, cimetidine	–
Hormones	Thyroxine, calcitonin, medroxyprogesterone	Epiphrine
Immunosuppressants	Tacrolimus, ciclosporin, interferon-alfa	Tacrolimus, ciclosporin
Methylxanthines	Theophylline, caffeine	–
Neuroleptics and dopamine depleters	Haloperidol, thioridazine, cinnarizine, reserpine, tetrabenazine	–

*Drugs known to cause postural and intention tremor (Morgan and Sethi, 2005)*.

A prominent intention tremor in the presence of eye-movement abnormalities, the presence of only an intention tremor, dysmetria, dyssynergia, trunk titubation, postural abnormalities, or hypotonia all suggest a cerebellar tremor (Degardin et al., 2012). Causes for cerebellar tremor include Friedreich’s ataxia, spinocerebellar ataxia syndromes, cerebellar infarction, multiple sclerosis, and Langerhans cell histiocytosis (Wnorowski et al., 2008; Perlman, 2011; Shneyder et al., 2011). In cerebellar tremor, different from ET, the tremor can even worsen after alcohol intake.

The differential diagnosis of tremor with atypical characteristics, such as an abrupt start and/or stop of tremor and tremor that lateralizes to one side, contains psychogenic tremor and intracranial tumors. Fluctuation in tremor during examination, an increase of tremor upon attention, decrease of tremor upon distraction, and entrainment of tremor to the frequency of repetitive movements all point toward psychogenic tremor, although the clinical characterization remains challenging (Edwards and Schrag, 2011; Schwingenschuh et al., 2011). Psychogenic tremor can occur in all positions, something not often seen in organic tremors. A useful test for discriminating psychogenic tremor from ET is distractibility (for example serial subtraction of 7 from 100 (sensitivity 72.7% specificity 73.3%) and tapping different fingers on to their thumbs in sequence (sensitivity 58.3%, specificity 84.4% Edwards and Schrag, 2011)). Contralateral tapping while stretching the affected limb is also a useful distraction task (Spiegel et al., 1998).

Some rare tremor disorders are associated with other signs, which can aid the diagnosis. A positive family history for early cognitive or neuropsychiatric deficits in males could suggest a diagnosis of fragile X-associated tremor/ataxia syndrome, a rare X-linked conditions characterized by ataxia and intellectual disability, where tremor and ataxia can manifest during middle age (Berry-Kravis et al., 2007). Cognitive problems are not always present during the development of tremor and can occur later in the disease (Brunberg et al., 2002). Tremor in fragile X-associated tremor/ataxia syndrome can be easily misdiagnosed as ET.

Neuropathic tremor occurs in association with peripheral neuropathies. Most frequent neuropathies associated with tremors are immune-mediated demyelinating and hereditary peripheral neuropathies. Often there are additional neurological symptoms present in these patients, mainly muscle weakness and sensory deficits (Pont-Sunyer et al., 2012). Characteristically, these patients present with action tremor, but rest tremor also occurs (Bain, 2007).

In patients with a (family) history of epileptic seizures, familial cortical myoclonic tremor has to be suspected. These patients may describe their tremor as shivering-like twitching of the fingers and hands (van Rootselaar et al., 2005). These movements occur mainly during posture and can also easily be misinterpreted as ET. Tremor in familial cortical tremor is in fact not a real “tremor”, but myoclonus mimicking tremor. Tremor recordings show a high burst frequency (up to 20 Hz) (van Rootselaar et al., 2005). Also, epilepsia partialis continua can give seemingly regular contractions in the hand, which can be confused with ET (Bien and Elger, 2008).

Finally, two tremor disorders are distinguished from other disorders by their frequency. Occurrence of a high-frequency tremor (>13 Hz) in the lower limbs that occurs or increases upon standing is suggestive of orthostatic tremor and is absent during rest. Patients usually complain of a feeling of unsteadiness during stance relieved by walking and sitting down, and do not mention tremor. With a stethoscope, the fast beating “helicopter sign” can often be heard (Gerschlager and Brown, 2011). A very low frequency tremor during rest (<4.5 Hz), which increases upon posture, and increases even further upon intentional movements, is suggestive for a Holmes’ (or rubral/midbrain) tremor (Deuschl et al., 1998; Seidel et al., 2009). Holmes’ tremor is often not as rhythmic as most other tremor disorders. Holmes tremor is usually caused by a lesion in the dopaminergic nigrostriatal or cerebello-thalamic pathways, and often accompanied by other neurological signs. A delay of 4 weeks to even 2 years has been described between the cause of the lesion (e.g., a cerebrovascular accident) and the occurrence of Holmes’ tremor (Deuschl et al., 1998).

### Documentation of tremor

**Figure 1:**
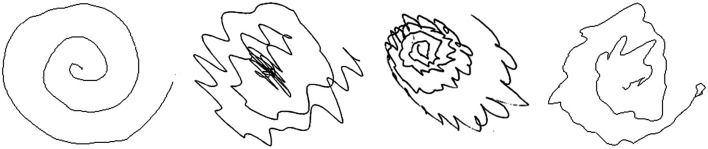
**Spiral drawings of (from left to right) a healthy control, a patient with ET, PD, and cortical tremor**.

In addition to the standard clinical examination, recording the drawing of an “Archimedes spiral” and the patients’ handwriting, can aid in the evaluation of disease progression and therapeutic response (Elble et al., 1990). Several studies have proven the clinical use of spiral drawing in ET (Elble et al., 1996; Louis et al., 2012). One study used spiral analysis to assess tremor severity and spiral diameter differences in ET and PD (Knoebel and Bain, 2009). ET patients had significantly severe tremor during spiral drawing, and PD patients drew spirals with significantly smaller diameters (Knoebel and Bain, 2009). When micrographia is present, the positive likelihood for PD increases between 2.8 and 5.9 times, the absence of micrographia gives a negative likelihood range of 0.30–0.44 (Rao et al., 2003). See Figure 1 for an example of the “Archimedes spiral” in several tremor disorders.

### Ancillary examinations

The extent to which a patient with tremor undergoes further ancillary examinations depends on the complexity of the case and whether a diagnosis can be established on patient history and clinical examination alone. Figure 4 provides an overview of the diagnostic work-up of a patient with tremor. Most ancillary examinations are directed at differentiating ET from PD, e.g., EMG and [^123^I]-FP-CIT single photon emission computerized tomography. It is recommended to test thyroid function routinely in patients with action tremor, or if there has been a recent unexplained exacerbation of tremor (Bain, 2007). Determining the serum TSH level is a sensitive and inexpensive marker to exclude tremor caused by hyperthyroidism (Beckett, 1995). Also, in unexplained tremor in patients under 55 years of age, Wilson disease should be excluded. A serum ceruloplasmin level lower than 50 mg/l is strong evidence for the diagnosis of Wilson disease. Modestly low serum ceruloplasmin needs further evaluation. Serum ceruloplasmin within the normal range does not exclude the diagnosis. Basal 24-h urinary excretion of copper can subsequently be determined if the diagnosis is uncertain. The 24-h copper excretion is typically >100 g in symptomatic patients, but finding >40 g may still indicate Wilson disease (Roberts and Schilsky, 2008). When in doubt, a 24-h urinary copper collection, pre- and post-penicillamine challenge, should be performed (Bain, 2007). MRI should be performed when Wilson disease is suspected (see Magnetic Resonance Imaging; Roberts and Schilsky, 2008).

A new and promising method, not further discussed in this review, for measuring tremor characteristics is currently being developed, with the help of in-built accelerometers of mobile phones. At the moment, the clinical relevance of these mobile phone applications is being investigated, but preliminary results are promising for differentiating several tremor disorders (Joundi et al., 2011; Saifee et al., 2012).

#### Electromyography

**Figure 2:**
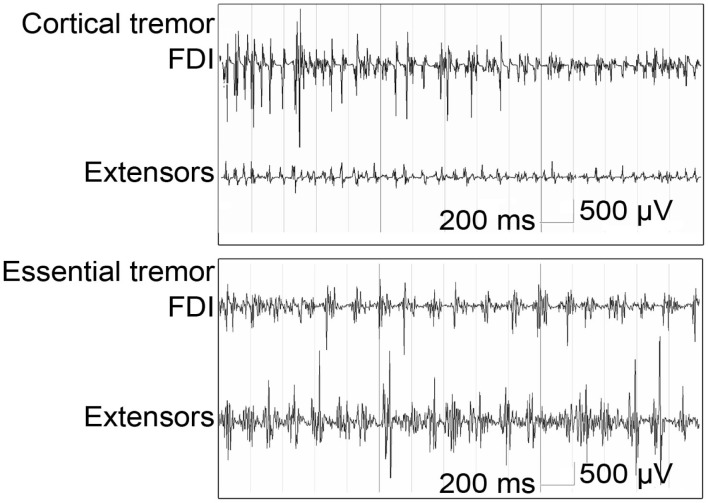
**Bipolar EMG from right first dorsal interosseous (FDI) and wrist extensors (Extensors) during posture in cortical tremor: high frequent bursts of <0.05 s (13–18 Hz) and essential tremor: rhythmic bursts at a frequency of approximately 6 Hz; burst duration is >0.05 s (figure adapted from van Rootselaar et al., 2006)**.

Electromyography is a simple and relatively inexpensive technique that can be very useful to support or establish a correct diagnosis (Benaderette et al., 2006). Figure 2 gives an example of an EMG recording in a patient with cortical tremor and a patient with ET.

Tremor frequency determined by EMG can be a discriminator when differentiating ET from PD. Both disorders show an overlap in frequency distributions, particularly in the 5.5–6 Hz range. A tremor frequency below 5.5 Hz suggests PD; a tremor frequency above 6 Hz suggests ET (Burne and Boljevac, 2002). A prospective study by Gironell posed a set of six neurophysiological criteria for ET which give a sensitivity of 97.7%, a specificity of 82.3%, a positive predictive value of 95.1% and a negative predictive value of 91.1% (Gironell et al., 2004), these criteria are summarized in Table 6. With these criteria, postural tremor in ET could successfully be distinguished from postural tremor in PD. Furthermore, in ET, agonist and antagonist muscles usually show synchronous activity, while in contrast, tremor in PD is often caused by alternating contraction of agonist and antagonist muscles (Nistico et al., 2011). Compared to ET, enhanced physiological tremor typically shows a higher frequency and shorter burst duration (Milanov, 2001). Milanov (2001) found cerebellar tremor to have a frequency around 9 Hz during posture and action, decreasing to 6 Hz upon intention. This contradicts with the frequency range stated by the Movement Disorders Society, which addresses cerebellar tremor as mainly intentional tremor below 5 Hz (Deuschl et al., 1998). In the study by Milanov et al., differentiating cerebellar and enhanced physiological tremor with solely EMG is challenging. The frequency of cerebellar tremor is generally lower than enhanced physiological tremor, but more importantly, the bursts are better defined in cerebellar tremor. Usually, the maximum amplitude in cerebellar tremor occurs during intentional movements, while in enhanced physiological tremor, intention tremor is rarely seen (Milanov, 2001).

**Table 6:** **Neurophysiological criteria for ET (Gironell et al., 2004)**.

Neurophysiological criteria for ET
1. Rhythmic burst of postural tremor on EMG
2. Tremor frequency ≥4 Hz
3. Absence of rest tremor, or, if present, frequency 1.5 Hz lower than the postural tremor
4. Absence of tremor latency from rest to postural position (>2 s)
5. Changes of the dominant frequency peak ≤1 Hz after the weight load test
6. No changes in tremor amplitude after mental concentration

Distinguishing between ET and dystonic tremor can be difficult. Compared to ET, patients with cervical dystonia and limb tremor showed greater tremor irregularity (up to 50% more) measured by cycle-to-cycle variability (Shaikh et al., 2008).

**Figure 3:**
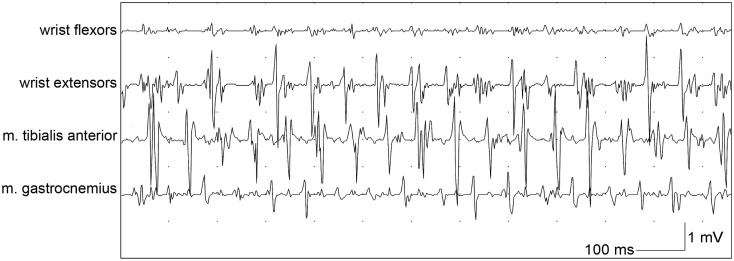
**Bipolar EMG of a patient with orthostatic tremor from wrist flexors, wrist extensors, tibialis anterior muscle and gastrocnemius muscle while leaning with both arms against a wall and standing, with a typical frequency of around 14 Hz**.

When orthostatic tremor is suspected, EMG is necessary to confirm the diagnosis, and shows a typical high-frequency (13–18 Hz) EMG pattern which appears after a short period of standing (orthostatism, Figure 3; Yague et al., 2012). Additionally, EMG signals in orthostatic tremor are highly coherent between left and right legs, with coherency values of up to 0.99. These high coherency values are rarely seen in other tremor disorders (Lauk et al., 1999).

Several tremor disorders show a change in frequency and/or amplitude upon loading of the stretched limb. In psychogenic tremor, an increase in amplitude during loading of the limb can be used to support the diagnosis, but its absence should not be used to exclude psychogenic tremor (an increase in amplitude of 130% gives a specificity 92% and a sensitivity of 33%; Schwingenschuh et al., 2011). ET is characterized by a tremor frequency not susceptible to changes upon loading, because of a fixed central oscillating mechanism. One of the proposed ET neurophysiological criteria by Gironell et al. (2004) is that the dominant frequency peak may not decrease by more than 1 Hz after loading. In enhanced physiologic tremor, the tremor frequency peak decreases with more than 1 Hz or a second tremor frequency peak at a lower frequency appears upon loading (Deuschl et al., 2001). No study on specificity and sensitivity for differentiating these patient groups is published. Elble (2003), however, found 8% of the healthy population to have an EMG pattern that is indistinguishable from mild ET.

Schwingenschuh et al. propose a set of EMG and accelerometry markers to be able to establish a positive diagnosis of psychogenic tremor, instead of a diagnosis of exclusion. A score of 3 or more suggests psychogenic tremor. Markers included “incorrect tapping performance at 1, 3, and 5 Hz (maximum three points), entrainment, suppression, or pathological frequency shift at 1, 3, and 5 Hz (maximum three points), pause or 50% reduction in amplitude of tremor with ballistic movements (one point), tonic co-activation before tremor onset (one point), coherence of bilateral tremors (one point), and increase of tremor amplitude with loading (one point)” (Schwingenschuh et al., 2011).

Electromyography in familial cortical tremor is used to confirm that the tremor is actually myoclonus, and shows periodic, irregular muscle bursts with a short burst duration of about 50 ms (van Rootselaar et al., 2005). In the case of suspected neuropathic tremor, nerve conduction studies should be performed with the help of EMG (Bain, 2007).

#### Long-term EMG recording

Using 10-h long continuous EMG recordings, Breit et al. developed a mathematical equation, based on mean tremor frequency, tremor occurrence (percentage of segments, in which tremor occurred), and phase (cross-spectral analysis of extensor and flexor EMG signals) to be able to differentiate ET from PD (Eq. 1).

$$\begin{array}{rcll} F & =20.9-\left(3.76\cdot \text{mean}\;\text{frequency}\right)+\left(\text{0}\text{.11}\cdot \text{tremor}\;\text{occurence}\right) & & \\ & \;-\left(\text{0}\text{.077}\cdot \text{Standard}\;\text{deviation}\;\text{of}\;\text{phase}\right) & & \text{(1)} \end{array}$$

Positive values of F predict the diagnosis of PD whereas negative values predict the diagnosis of ET. This equation was applied on 13 patients in early stages of the disease, and yielded a 100% fit between diagnosis predicted by long-term EMG and the diagnoses inferred by SPECT imaging (Breit et al., 2008).

#### Coherence analysis (EMG-EEG)

Little is published on the diagnostic value of coherence analysis in differentiating tremor syndromes. Simultaneous EMG-EEG can be used to look for cortico-muscular coherences at the tremor frequency. Hellwig et al. (2001) found cortico-muscular coherences at the tremor frequency in five out of nine arms in patients with essential tremor and were unable to find this coherence in patients with enhanced physiological tremor. Van Rootselaar et al. used coherence analysis to differentiate cortical tremor from essential tremor. In a group of patients with “Familial Cortical Myoclonic Tremor with Epilepsy” patients, a strong cortico- and intermuscular coherence in the 8- to 30-Hz range was shown with EEG preceding EMG, this coherence was not found in essential tremor and healthy controls (van Rootselaar et al., 2006).

#### Somatosensory evoked potential measurements

Patients with cortical tremor, presenting with tremulous movements, sometimes resembling ET, and with or without a (family) history of epilepsy, can show a so-called “giant potential” upon median nerve stimulation during a somatosensory evoked potential measurement (Okuma et al., 1997; van Rootselaar et al., 2002). A giant potential is in line with cortical hyperexcitability and a sign of cortical myoclonus.

#### [^123^I]-FP-CIT single photon emission computerized tomography

[^123^I]-FP-CIT single photon emission computerized tomography, also called (DAT-) SPECT imaging or FP-CIT-SPECT imaging, can be used to assess nigrostriatal denervation, a sign of PD (Benamer et al., 2000). Vlaar et al. (2007) performed a meta-analysis on the diagnostic accuracy of SPECT imaging in parkinsonian syndromes. They found SPECT imaging with presynaptic tracers (such as ^123^I-ioflupane) to be highly accurate to differentiate between PD and ET (sensitivity 80–100%, specificity 80–100%). One study showing lowest specificity included not only patients with ET, but also patients with isolated postural tremor and postural tremor in combination with rest tremor (Lee et al., 1999). In a more recent study, Coria et al. also used SPECT to differentiate ET and PD. Only patients with an isolated action tremor were included, without resting tremor, bradykinesia, or other hypokinetic parkinsonian symptoms. They found reduced striatal uptake in 68.3% of included patients. The odds ratio of finding reduced striatal uptake was increased three times for patients aged over 50, and increased five times for patients with an asymmetrical action tremor (Coria et al., 2012). However, there are also studies that found reduced striatal uptake in ET compared to controls, but less severely then PD (Isaias et al., 2008). None of these ET patients had clinical signs of PD, which makes the clinical use of these findings questionable. In another study comparing SPECT scans in parkinsonian syndromes with non-parkinsonian syndromes, three patients with atypical asymmetrical postural tremor, initially diagnosed with ET and a fourth patient with a 6-month history of gait ataxia, slight bradykinesia and rigidity, cerebellar tremor, and frequent falls, initially diagnosed with cerebellar tremor had an abnormal SPECT scan. Their diagnosis was altered in subsequently PD and multiple system atrophy. They concluded that SPECT studies may act as an adjunct to diagnosis (Bairactaris et al., 2009). One study looked at nine patients referred with suspected psychogenic parkinsonism. SPECT imaging in this study was successful in differentiating pure psychogenic parkinsonism from psychogenic parkinsonism plus PD, and supported the diagnosis of underlying PD in five of nine patients (Benaderette et al., 2006).

An Italian cost-effectiveness study showed that using SPECT imaging for differentiating unclear cases of ET from PD is cost-effective because of decreasing time on potentially beneficial treatment at a lower overall cost (Antonini et al., 2008). The United Kingdom National Institute for Health and Clinical Excellence guideline also suggests the use of SPECT imaging in patients with tremor where ET cannot be clinically differentiated from PD (Stewart, 2007). SPECT could be used to avoid the costs of treating people who do not suffer from PD. However, they also advise not to use SPECT in all people with PD in place of initial clinical examination.

In the last decade, SPECT imaging has been used as a surrogate marker for disease progression. An unexpected consequence of this was that around 10% of patients, who initially fulfilled the clinical diagnostic criteria of PD, had normal nigrostriatal uptake (Whone et al., 2003). These patients have been referred to as “SWEDDs” (Scans Without Evidence of Dopaminergic Deficit). A study comparing clinical and neurophysiological characteristics of SWEDDs with PD and other tremor disorders by Schwingenschuh et al. suggested that these patients share characteristics with adult-onset dystonic tremor. Furthermore, these SWEDDs patients did not show true bradykinesia (with fatiguing and decrement) and did not respond to levodopa treatment (Schwingenschuh et al., 2010).

#### Acute levodopa challenge test

Many patients with PD respond to a single dose of levodopa, and therefore this test is regularly used in clinical practice to differentiate ET from PD. Using levodopa response as a diagnostic test has so far only been studied for differentiating parkinsonian syndromes. One systematic review that included studies with *de novo* PD and well-established PD found acute levodopa treatment (125–275 mg) to have a positive predictive value of 0.69 (95% CI 0.59 to 0.90; Clarke and Davies, 2000). The acute levodopa challenge consisted of a standard dose of 275 mg levodopa plus decarboxylase inhibitor. Most challenges were performed during a day admission after domperidone pre-treatment. Response to chronic levodopa treatment gave a positive predictive value of 0.76 (95% CI 0.70 to 0.82). A chronic levodopa treatment consisted of a maximum dose of 1000 mg with a duration of treatment varying from 1 to 6 months (Clarke and Davies, 2000). However, this has not been assessed for differentiating ET from PD. Also, the effect of a levodopa trial on tremor has not been assessed separately.

#### Transcranial sonography

Transcranial sonography can detect increased midbrain echogenicity (Berg et al., 2005). In a study by Bartova, sensitivity and specificity for transcranial sonography in patients with PD, parkinsonian syndromes, ET, and psychogenic movement disorders were evaluated. Transcranial sonography and SPECT findings correlated in 84% of patients (sensitivity 89.7%, specificity 60% for transcranial sonography, sensitivity 96.6%, specificity 70% for SPECT imaging; Bartova et al., 2012). In this study, the diagnostic accuracy of transcranial sonography was comparable to the more expensive SPECT imaging. Gaenslen et al. (2008) found a sensitivity of 90.7% and a specificity of 82.4%. Berg et al. performed a 5-year follow up study of PD cases after transcranial sonography. They found no significant changes in sonography findings across time. It is suggested that the presence of midbrain hyperechogenicity is a trait rather than a state marker for susceptibility to PD (Berg et al., 2005). In a study by Berg et al. (1999), 90% of PD patients exhibited midbrain hyperechogenicity and 8.6% of the healthy population exhibited the same ultrasound signal, associated in more than 60% with a functional deficit of the nigrostriatal system as detected by 18 F-labeled dopa positron emission tomography (PET) examinations. Transcranial sonography, although being much less expensive compared to SPECT imaging, requires high expertise and is to a certain extent, examiner-dependent. Moreover results need to be further investigated. For these reasons, its clinical use remains a subject of debate. However, in settings were SPECT imaging is not available, transcranial sonography can prove to be useful in unclear cases were PD is suspected and where an experienced investigator is available.

#### Magnetic Resonance Imaging

Hyperintensities on T2 MRI in the region of the basal ganglia can be seen in Wilson disease. According to the American Association for the Study of Liver Diseases: “MRI should be considered in Wilson disease prior to treatment in all patients with neurological symptoms and be part of the evaluation of any patient presenting with neurological symptoms consistent with Wilson disease” (Roberts and Schilsky, 2008).

Fragile X-associated tremor/ataxia syndrome in symptomatic males shows a characteristic pattern of MRI findings. This pattern includes increased T2 signal intensity in the middle cerebellar peduncle and deep white matter of the cerebellum medial, superior, and inferior to the dentate nuclei (Brunberg et al., 2002).

In patients with spinocerebellar ataxia, three patterns of damage can be seen on conventional MRI: spinal atrophy, olivopontocerebellar atrophy, and cortical cerebellar atrophy (Mascalchi, 2008). There is even a correlation between the specific pattern of changes on the MRI and different diseases underlying the spinocerebellar ataxia (Mascalchi, 2008).

In Holmes’ tremor, data on the relevance of MRI are sparse. In a case series of 10 patients, the structural lesion was due to hemorrhage in six patients and due to cerebral ischemia in four patients. The thalamus was lesioned in five cases, in other cases, involvement of the midbrain tegmentum, superior cerebellar peduncle, substantia nigra, pons, rubro-olivocerebello-rubral loop, rubro-spinal fibers and nigrostriatal fibers was seen (Gajos et al., 2010).

#### Olfactory tests

Olfactory dysfunction is found in about 80% of PD cases, regardless of disease stage or duration (Doty et al., 1988; Double et al., 2003). In a study comparing olfactory function of PD with ET using the University of Pennsylvania Smell Identification Test (UPSIT), the olfactory function of PD was significantly worse compared to ET (Shah et al., 2008). Using a cut-off score of 25, the sensitivity of the UPSIT was 83% and the specificity 94%. Raising the cut-off score to 30, improves the sensitivity to 97%, but reduces specificity (87%). In a study comparing olfactory function in ET with healthy controls, ET patients also showed significantly lower UPSIT scores (UPSIT for ET 29.0 ± 6.1 vs. 31.9 ± 4.6 in controls, *p* = 0.02), the UPSIT scores were not correlated with tremor severity or duration (Louis et al., 2002). In this study, 27% of ET cases had severe olfactory dysfunction (UPSIT cut-off score of 25), compared to 2.7% of controls. At present, olfactory tests can be helpful in the differentiation between PD and ET, but diagnosis should not rely solely on olfactory function. Therefore the clinical relevance of this test remains debatable.

## Conclusion

**Figure 4:**
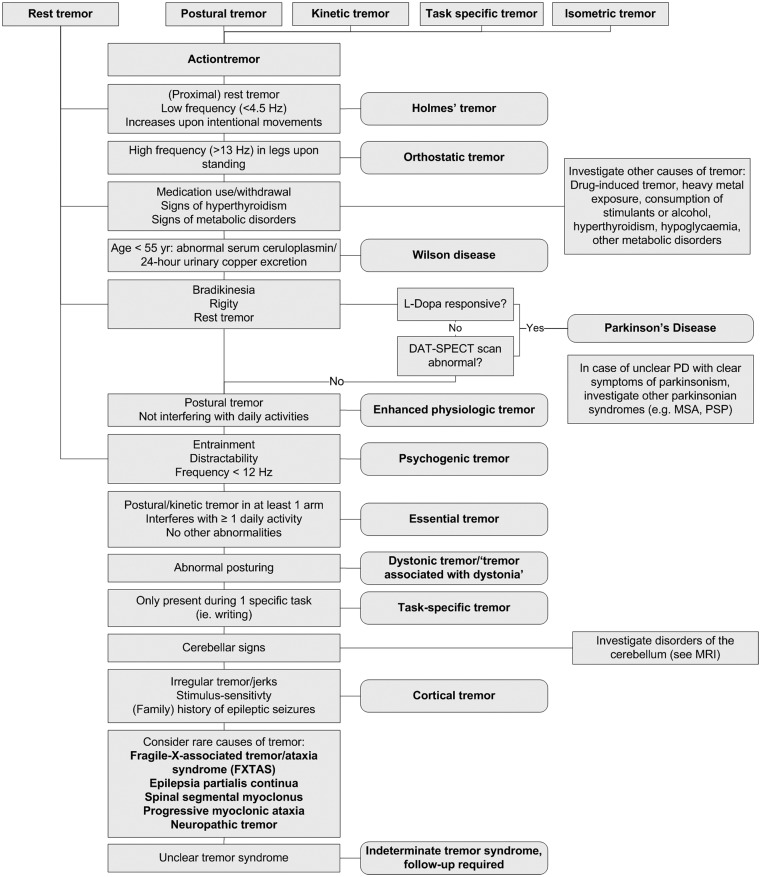
**Diagnostic work-up of a patient with tremor**.

In most patients, history and clinical examination are sufficient to establish a correct diagnosis, and further ancillary examinations will not be needed. Investigation should always be guided by tremor type(s) present and other associated signs and symptoms. Adherence to the diagnostic work-up provided in Figure 4 will help the diagnostic process of these patients. However, there are unclear cases, in which tremor disorders are notoriously difficult to differentiate. In these unclear cases, there are several techniques, including neurophysiological techniques and imaging, which can be useful. Unfortunately, many techniques have not been studied in large prospective, diagnostic studies to be able to determine important variables like sensitivity and specificity, and consequently, the diagnostic process in this patient group is often based more on empirical evidence than on quantitative studies.

## Conflict of Interest Statement

The authors declare that the research was conducted in the absence of any commercial or financial relationships that could be construed as a potential conflict of interest.
